# Deep learning-based classification of multiple fundus diseases using ultra-widefield images

**DOI:** 10.3389/fcell.2025.1630667

**Published:** 2025-07-17

**Authors:** Ming-Ming Duan, Xiang Tu

**Affiliations:** ^1^ Department of ophthalmology, JiuJiang City Key Laboratory of Cell Therapy, Jiujiang No. 1 People’s Hospital, JiuJiang, Jiangxi, China; ^2^ Department of Otolaryngology, The Seventh Affiliated Hospital, Sun Yat-sen University, ShenZhen, Guangdong, China

**Keywords:** deep transfer learning, ultra-widefield fundus images, retinal diseases, DenseNet121, XGBoost, multiple fundus diseases

## Abstract

**Purpose:**

This study aimed to develop a hybrid deep learning model for classifying multiple fundus diseases using ultra-widefield (UWF) images, thereby improving diagnostic efficiency and accuracy while providing an auxiliary tool for clinical decision-making.

**Methods:**

In this retrospective study, 10,612 UWF fundus images were collected from the JiuJiang No. 1 People’s Hospital and the Seventh Affiliated Hospital, Sun Yat-sen University between 2020 and 2025, covering 16 fundus diseases, including normal fundus, nine common eye diseases, and six rare retinal conditions. The model employed DenseNet121 as a feature extractor combined with an XGBoost classifier. Gradient-weighted Class Activation Mapping (Grad-CAM) was used to visualize the model’s decision-making process. Performance was evaluated on validation and external test sets using accuracy, recall, precision, F1 score, and AUC-ROC. The model’s diagnostic accuracy was also compared with that of junior and intermediate ophthalmologists.

**Results:**

The model demonstrated exceptional diagnostic performance. For common diseases such as retinal vein occlusion, age-related macular degeneration, and diabetic retinopathy, AUC values exceeded 0.975, with accuracy rates above 0.980. For rare diseases, AUC values were above 0.970, and accuracy rates surpassed 0.998. Grad-CAM visualizations confirmed that the model’s focus areas aligned with clinical pathological features. Compared to ophthalmologists, the model achieved significantly higher accuracy across all diagnostic tasks.

**Conclusion:**

The proposed deep learning model can automatically identify and classify multiple ophthalmic diseases using UWF images. It holds promise for enhancing clinical diagnostic efficiency, assisting ophthalmologists in optimizing workflows, and improving patient care quality.

## Introduction

With the rapid growth of the global elderly population, many age-related health issues have become increasingly prominent, among which the incidence of retinal diseases has significantly increased (Wong et al., 2014; Flaxman et al., 2017). As a vital element in vision formation, the retina directly impacts visual acuity. Therefore, the prompt, efficient, and precise detection of retinal lesions is of utmost importance. Traditional fundus photography is confined to the central 30–60 degrees of the retina, allowing identification of only the posterior pole (Shoughy et al., 2015). In contrast, Ultra-Wide-Field (UWF) fundus imaging offers a comprehensive view of nearly the entire retina with a 200° field of view, covering both the posterior pole and peripheral areas (Nagiel et al., 2016; Silva et al., 2012). This extensive coverage provides richer clinical information, aiding in the early diagnosis and timely intervention of conditions such as pathologic myopia, retinal vein occlusion, retinal detachment, retinal holes, diabetic retinopathy, and age-related macular degeneration (Tan et al., 2019; Ohsugi et al., 2017; Masumoto et al., 2019; Zhang et al., 2021; Horie and Ohno-Matsui, 2022). However, despite the fact that UWF technology can provide a broader coverage of the retina, doctors still face many challenges in identifying lesion features in these images. For instance, in the diagnosis and management of diabetic retinopathy, UWF fundus imaging can detect peripheral lesions that are difficult to find with traditional methods, which helps to more accurately assess the severity of the disease and formulate treatment strategies (Ashrafkhorasani et al., 2024; Silva et al., 2013). However, due to the complexity and large amount of information in UWF fundus images, doctors need to possess higher professional skills and experience when interpreting these images (Ghasemi Falavarjani et al., 2017).

In recent years, with the rapid development of artificial intelligence (AI) technology, its application in ophthalmic imaging examinations has become increasingly widespread, especially achieving remarkable progress in fields such as optical coherence tomography (OCT) (Gan et al., 2023a; Wei et al., 2023), fundus photography (Li et al., 2024; Rajalakshmi et al., 2018), and fluorescein fundus angiography (FFA) (Long et al., 2025; Wei et al., 2025). It is capable of rapidly and precisely identifying and analyzing the characteristics of fundus lesions, thereby enabling early screening, diagnosis stratification, and disease monitoring for various fundus diseases, which substantially enhances diagnostic efficiency and accuracy (Grassmann et al., 2018; Li et al., 2023). In previous studies, Nguyen et al. (2024) proposed a deep learning-based approach for retinal disease diagnosis using ultra-wide-field fundus images, achieving high accuracy with models like ResNet152. Despite their promising results, their work was limited to binary classification (normal vs. abnormal). In contrast, DenseNet121, with its unique dense connection mechanism and feature reuse capability, demonstrates significant advantages in handling complex medical image tasks. Its dense connection structure not only enhances feature propagation and reduces the problem of gradient vanishing, but also significantly improves parameter utilization efficiency and reduces model complexity through feature reuse, while enhancing the model’s generalization ability (Zhang et al., 2025). XGBoost, with its powerful classification performance and nonlinear combination ability, can further optimize classification results (Ye et al., 2022; Dong et al., 2018).

Therefore, this study proposes a hybrid deep learning model based on DenseNet121 and XGBoost for the intelligent diagnosis of 16 retinal conditions (including normal fundus, nine common eye diseases, and six rare diseases) in ultra-wide-field fundus images. The model innovatively combines the multi-scale feature extraction advantages of DenseNet121s dense connection architecture with XGBoost’s ensemble learning capabilities for handling imbalanced data (Gan et al., 2023b). By leveraging the complementary strengths of deep convolutional features and gradient boosting decision trees, it effectively addresses addressed the performance bottleneck of traditional end-to-end CNN models in classifying rare diseases.

## Methods

### Data collection

**FIGURE 1:**
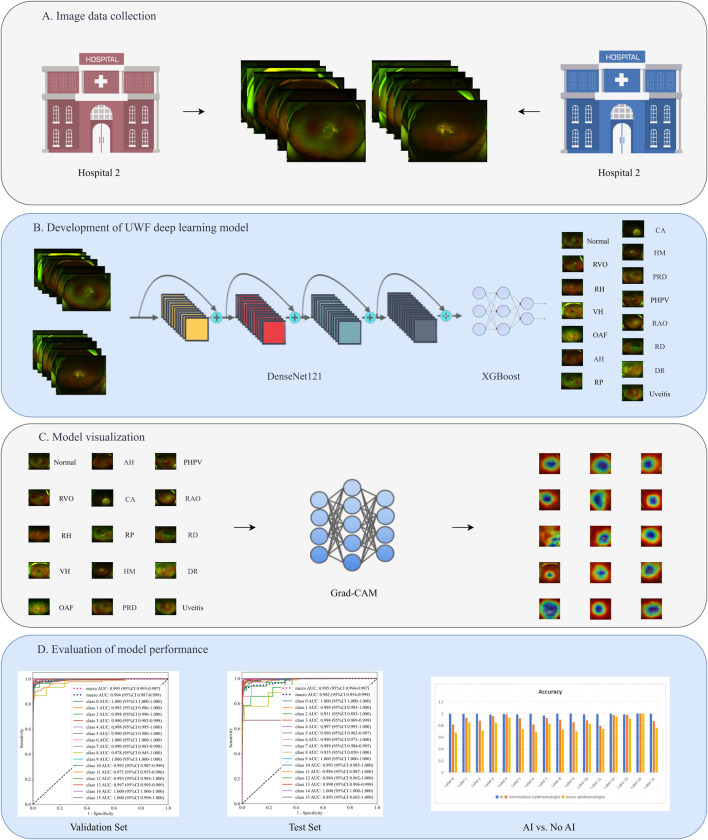
The study design flowchart. **(A)** Image data collection. **(B)** Development of UWF deep learning model. **(C)** Model visualization. **(D)** Evaluation of model performance.

This retrospective study collected ultra-widefield (UWF) fundus photographs from patients who visited the JiuJiang No. 1 People’s Hospital between January 2020 and January 2025. The dataset was randomly partitioned into training (80%) and validation (20%) sets. An additional external test set was compiled from UWF images obtained from the Seventh Affiliated Hospital, Sun Yat-sen University during the same period. The study protocol received approval from the institutional review boards of both participating hospitals and complied with the Declaration of Helsinki. As all patient data were anonymized and contained no personally identifiable information, the ethics committees waived the requirement for individual informed consent. The study design is illustrated in Figure 1.

### Image preprocessing

Initial quality control excluded images with poor resolution, excessive blurring, or duplicate content. Diagnostic labeling followed a rigorous two-stage process: 1) preliminary classification by an independent ophthalmologist using comprehensive clinical data from electronic medical records, and 2) validation by a senior retinal specialist who reviewed all ambiguous cases to ensure diagnostic consistency. Based on clinical diagnoses, images were categorized into 16 classes: Retinal Artery Occlusion (RAO, 0), Retinal Vein Occlusion (RVO, 1), Age-related Macular Degeneration (AMD, 2), Diabetic Retinopathy (DR, 3), Retinal Detachment (RD, 4), High Myopia (HM, 5), Uveitis (6), Vitreous Hemorrhage (VH, 7), Asteroid Hyalosis (AH, 8), Choroidal Atrophy (CA, 9), Peripheral Retinal Degeneration (PRD, 10), Retinal Hole (RH, 11), Retinitis Pigmentosa (RP, 12), Normal fundus (13), Ocular Albinism Fundus (OAF, 14), and Persistent Hyperplastic Primary Vitreous (PHPV, 15).

## Development of deep learning model

We employed DenseNet121, pretrained on ImageNet, for feature extraction. The model’s dense connection architecture facilitates feature propagation and reuse, enhancing its ability to capture both local and global image features. All images were resized to 224 × 224 pixels to meet network input requirements. To improve model generalization and robustness, we implemented data augmentation techniques including random flipping, rotation (±30°), and contrast-limited adaptive histogram equalization (CLAHE) (Wu et al., 2022).

To address class imbalance, we applied stratified sampling to maintain consistent class distributions across training, validation, and test sets. Rare disease samples underwent 20-fold augmentation (rotation, horizontal flipping, CLAHE), yielding 300 effective training samples per rare disease category. The model incorporated Focal Loss in DenseNet121 to reduce majority class weighting, while the XGBoost classifier used scale_pos_weight (reciprocal of class frequency) to further mitigate imbalance effects.

## Computational implementation

Model training and evaluation were conducted on a workstation equipped with an NVIDIA RTX 4090 GPU (24 GB VRAM), Intel Core i9-13900K CPU, and 64 GB DDR5 RAM, achieving an average inference speed of 0.07 ± 0.01 s per image. Training parameters included:

• Batch size: 32
• Initial learning rate: 0.001 (reduced by factor of 0.1 every 50 epochs)
• Optimizer: Adam (β1 = 0.9, β2 = 0.999)
• Training epochs: 150 (with early stopping if validation loss failed to improve for 15 consecutive epochs)

High-level features were extracted from the “features.norm5” layer prior to global average pooling. These features underwent dimensionality reduction via principal component analysis (PCA) before being classified by a tuned XGBoost model (Gan et al., 2023a).

### Model visualization

After the completion of model training, we employ the Gradient-weighted Class Activation Mapping (Grad-CAM) (Selvaraju et al., 2024) technique to visualize the decision-making basis of the model. Specifically, through backpropagation, we compute the gradient of the target category with respect to the feature map of the last convolutional layer in DenseNet121. These gradients are then multiplied element-wise by the corresponding feature maps and aggregated via weighted summation to generate a normalized activation map. Subsequently, this activation map is transformed into a heatmap and overlaid onto the original UWF fundus image, thereby visually highlighting the regions of interest that the model focuses on. Then, a specialist in retinal diseases from a tertiary hospital evaluated the consistency between the heat map-marked regions and the corresponding clinical pathological features. This process provides valuable insights into the model’s decision-making process, offering interpretable visualization support.

### Comparison of diagnostic performance between ophthalmologists and AI models

Diagnostic performance was compared between the AI model and:

1. Junior ophthalmologists (<5 years’ experience, completed residency training)
2. Intermediate ophthalmologists (5–10 years’ experience, attending physicians)

Evaluations used a standardized electronic platform allowing image parameter adjustment but no clinical context. A single-blind design ensured objective assessment, with clinicians unaware of ground truth labels.

### Statistical analysis

All analyses were performed using Python 3.9.7. Model performance was assessed using:

1. Accuracy: Overall classification correctness
2. Recall: True positive detection rate
3. Precision: Positive predictive value
4. F1 score: Harmonic mean of recall and precision
5. AUC-ROC: Classification discrimination across thresholds

ROC curves visualized true-positive vs. false-positive tradeoffs, while confusion matrices detailed per-class prediction errors. Comparative analyses with clinician performance provided practical insights into clinical applicability.

## Results

### Dataset characteristics

The dataset of this study contains a total of 10,612 ultra-wide-angle fundus images, covering 16 different types of retinal images, which were divided into a training set (6,980 images), a validation set (1,820 images), and an external test set (1,812 images). The dataset includes normal retinal images as well as images of 9 common eye diseases (RVO, AMD, DR, RD, HM, Uveitis, VH, PRD, RH) and 6 rare retinal diseases (RAO, AH, CA, RP, OAF, PHPV). The distribution of images of each category in the training set, validation set, and external test set are detailed in Table 1.

**TABLE 1:** Characteristics of the datasets.

Image category	Training set	Vlidation set	External test set
RAO	11	4	3
RVO	333	85	89
MD	67	14	18
DR	1002	246	215
RD	1158	273	298
HM	586	144	141
Uveitis	38	7	3
VH	1243	298	309
AH	49	9	15
CA	21	6	5
PRD	93	104	113
RH	325	83	88
RP	108	32	29
Normal	1906	508	476
OAF	15	4	3
PHPV	25	3	7

RAO: retinal artery occlusion; RVO: retinal vein occlusion; AMD: Age-related Macular Degeneration; DR: diabetic retinopathy; RD: retinal detachment; HM: high myopia; VH: vitreous hemorrhage; AH: asteroid hyalosis; CA: choroidal atrophy; PRD: peripheral retinal degeneration; RH: retinal hole; RP: retinitis pigmentosa; OAF: ocular albinism fundus; PHPV: persistent hyperplastic primary vitreous.

### Evaluation of model diagnostic performance

Our deep learning model has exhibited exceptional performance in diagnosing nine common eye diseases (RVO, AMD, DR, RD, HM, Uveitis, VH, PRD, and RH). On the validation set, the AUC values for all diseases exceeded 0.975, with accuracy rates consistently above 0.980. Notably, the AUC values for RVO, AMD, DR, RD, HM, Uveitis, PRD, and RH reached or exceeded 0.990, while the accuracy rates for AMD, RD, Uveitis, and RH surpassed 0.990. These results underscore the model’s high precision and robust discriminative ability in identifying these prevalent ocular conditions. On the external test set, the model maintained strong performance, achieving AUC values above 0.950 and accuracy rates exceeding 0.970. This further validates its reliability and stability in real-world applications.

**FIGURE 2:**
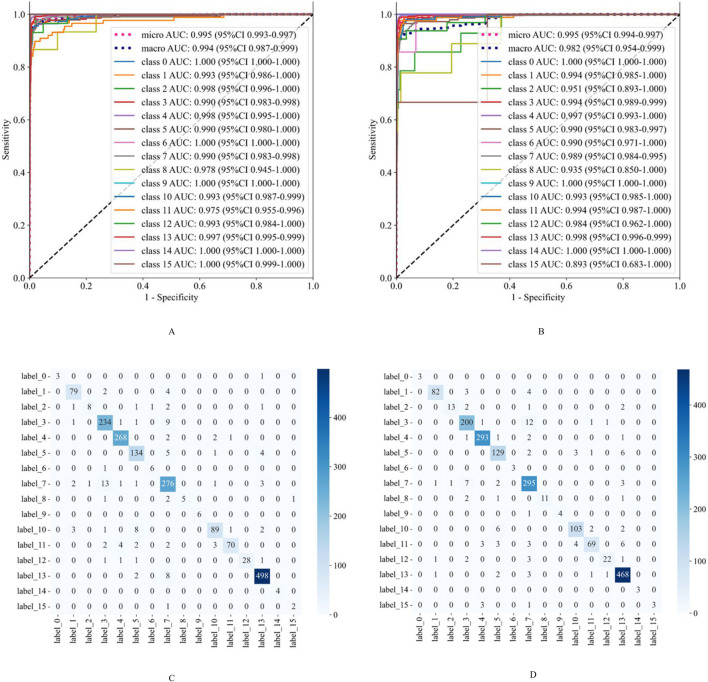
The classification performance evaluation results of the model on the validation set and the external test set. **(A,B)** are the ROC curves of the model on the validation set and the external test set, respectively. **(C,D)** are the confusion matrices of the model on the validation set and the external test set, respectively. The rows of the confusion matrix represent the actual categories, and the columns represent the predicted categories. Class 0: Retinal Artery Occlusion (RAO); Class 1: Retinal Vein Occlusion (RVO); Class 2: Age-related Macular Degeneration (AMD); Class 3: Diabetic Retinopathy (DR); Class 4: Retinal Detachment (RD); Class 5: High Myopia (HM); Class 6: Uveitis; Class 7: Vitreous Hemorrhage (VH); Class 8: Asteroid Hyalosis (AH); Class 9: Choroidal Atrophy (CA); Class 10: Peripheral Retinal Degeneration (PRD); Class 11: Retinal Hole (RH); Class 12: Retinitis Pigmentosa (RP); Class 13: Normal; Class 14: Ocular Albinism Fundus (OAF); Class 15: Persistent Hyperplastic Primary Vitreous (PHPV).

For six rare retinal diseases, the model also demonstrated superior diagnostic capabilities. On the validation set, the AUC values for these diseases were all above 0.970, with accuracy rates reaching as high as 0.998. On the external test set, the AUC values remained above 0.893, and the accuracy rates exceeded 0.991. Despite the relatively small sample size of rare diseases, which may pose challenges to the model’s generalization, its performance remained consistently high. This highlights the model’s significant potential and reliability in diagnosing rare retinal diseases. The detailed diagnostic performance indicators of the model are presented in Table 2, and the ROC curves and confusion matrices for different types of retinal diseases are shown in Figure 2.

**TABLE 2:** The performance metrics of the model on the validation set and the external test set.

Model	AUC (95%CI)	Accuracy	Precision	Recall	Specificity
Validation set					
RAO	1.000 (1.000–1.000)	0.999	1.000	0.75	1.000
RVO	0.993 (0.986–1.000)	0.993	0.919	0.929	0.996
AMD	0.998 (0.996–1.000)	0.996	0.889	0.571	0.999
DR	0.990 (0.983–0.998)	0.982	0.918	0.951	0.987
RD	0.998 (0.995–1.000)	0.993	0.975	0.982	0.995
HM	0.990 (0.980–1.000)	0.986	0.893	0.931	0.990
Uveitis	1.000 (1.000–1.000)	0.999	0.857	0.857	0.999
VH	0.990 (0.983–0.998)	0.969	0.887	0.926	0.977
AH	0.978 (0.945–1.000)	0.998	1.000	0.556	1.000
CA	1.000 (1.000–1.000)	1.000	1.000	1.000	1.000
PRD	0.993 (0.987–0.999)	0.988	0.927	0.856	0.996
RH	0.975 (0.955–0.996)	0.992	0.972	0.843	0.999
RP	0.993 (0.984–1.000)	0.998	1.000	0.875	1.000
Normal	0.997 (0.995–0.999)	0.988	0.976	0.980	0.991
OAF	1.000 (1.000–1.000)	1.000	1.000	1.000	1.000
PHPV	1.000 (0.999–1.000)	0.999	0.667	0.667	0.999
External test set					
RAO	1.000 (1.000–1.000)	1.000	1.000	1.000	1.000
RVO	0.994 (0.985–1.000)	0.994	0.965	0.921	0.998
AMD	0.951 (0.893–1.000)	0.997	0.929	0.722	0.999
DR	0.994 (0.989–0.999)	0.982	0.922	0.930	0.989
RD	0.997 (0.993–1.000)	0.993	0.977	0.983	0.995
HM	0.990 (0.983–0.997)	0.985	0.896	0.915	0.991
Uveitis	0.990 (0.971–1.000)	1.000	1.000	1.000	1.000
VH	0.989 (0.984–0.995)	0.975	0.902	0.955	0.979
AH	0.935 (0.850–1.000)	0.998	1.000	0.733	1.000
CA	1.000 (1.000–1.000)	0.999	1.000	0.800	1.000
PRD	0.993 (0.985–1.000)	0.991	0.936	0.912	0.996
RH	0.994 (0.987–1.000)	0.987	0.932	0.784	0.997
RP	0.984 (0.962–1.000)	0.995	0.917	0.757	0.999
Normal	0.998 (0.996–0.999)	0.983	0.955	0.983	0.984
OAF	1.000 (1.000–1.000)	1.000	1.000	1.000	1.000
PHPV	0.893 (0.683–1.000)	0.998	1.000	0.429	1.000

RAO: retinal artery occlusion; RVO: retinal vein occlusion; AMD: Age-related Macular Degeneration; DR: diabetic retinopathy; RD: retinal detachment; HM: high myopia; VH: vitreous hemorrhage; AH: asteroid hyalosis; CA: choroidal atrophy; PRD: peripheral retinal degeneration; RH: retinal hole; RP: retinitis pigmentosa; OAF: ocular albinism fundus; PHPV: persistent hyperplastic primary vitreous.

### Grad-CAM visualization

**FIGURE 3:**
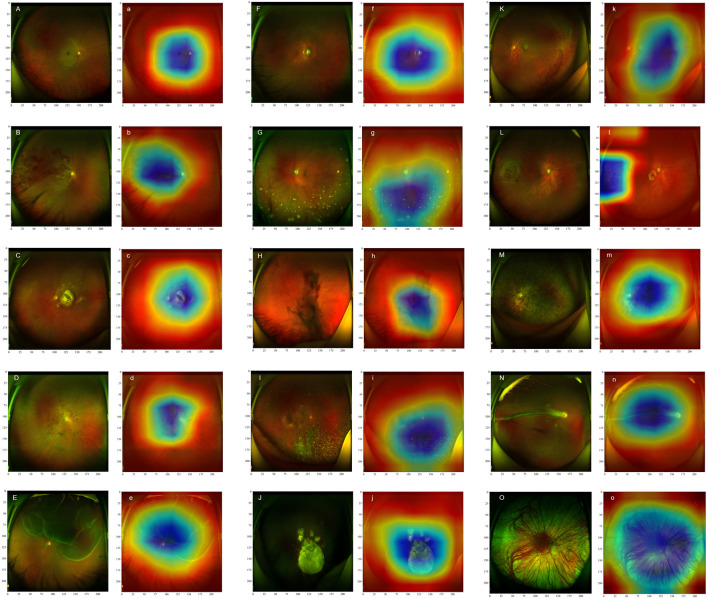
Ultra-Wide-Field (UWF) fundus images of different retinal diseases (marked with capital letters) and their corresponding heat maps (marked with lowercase letters) are presented. The heat maps illustrate the significance of image features for the DenseNet121 model, with different colors indicating the extent to which the model focuses on these features during classification. Specifically, blue areas highlight the regions that receive the greatest attention from the model during classification, whereas red areas correspond to regions with relatively lower attention. Retinal Artery Occlusion **(A,a)**: Retinal Vein Occlusion **(B,b)**; Age-related Macular Degeneration **(C,c)**; Diabetic Retinopathy **(D,d)**; Retinal Detachment **(E,e)**; High Myopia **(F,f)**; Uveitis **(G,g)**; Vitreous Hemorrhage **(H,h)**; Asteroid Hyalosis **(I,i)**; Choroidal Atrophy **(J,j)**; Peripheral Retinal Degeneration **(K,k)**; Retinal Hole **(L,l)**; Retinitis Pigmentosa **(M,m)**; Ocular Albinism Fundus **(N,n)**; Persistent Hyperplastic Primary Vitreous **(O,o)**.

This study utilized the Grad-CAM technique to visually interpret the decision-making logic of the model. Its clinical diagnostic value has been strictly verified by ophthalmologists specializing in retinal diseases in tertiary hospitals. As shown in Figure 3, the visualization results exhibit a high degree of consistency with the typical pathological features of various retinal diseases. For retinal vascular diseases, the heat map of RVO highlighted the abnormally dilated venous vessels, whereas that of RAO emphasized the characteristic cherry red spot. In macular diseases, the significant regions of AMD were predominantly distributed in the lesion areas of the macula. The hotspots of DR exhibited prominent pathological features such as microaneurysms, hemorrhage, and hard exudates. The heat map of RD was prominently concentrated in the neuroepithelial layer detachment region. For HM, the feature activation regions were localized in the optic disc and choroidal atrophy lesions. The visualization results of peripheral retinal degeneration and holes accurately pinpointed the areas of retinal tissue defects. The heat distribution of uveitis was predominantly concentrated in the inflammatory exudation area. The significant mapping of VH precisely marked the suspended blood clots. The heat map of RP focused on the areas with dense pigment deposition, while the activation regions of CA were located in the thinned choroid. The area of interest for OAF corresponded to abnormal vessel regions, and the heat distribution of PHPV aligned accurately with the residual embryonic vitreous tissue. This high consistency with clinical pathological features substantiated the medical rationality of the model’s diagnostic decisions.

### Comparison of diagnostic performance between ophthalmologists and AI models

**FIGURE 4:**
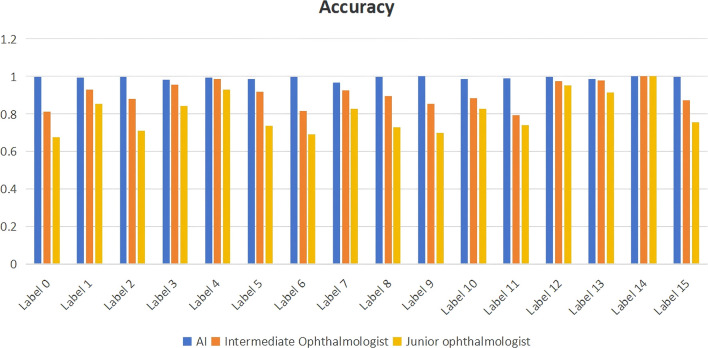
Comparison of diagnostic performance between ophthalmologists of different levels and artificial intelligence models. label 0: Retinal Artery Occlusion (RAO); label 1: Retinal Vein Occlusion (RVO); label 2: Age-related Macular Degeneration (AMD); label 3: Diabetic Retinopathy (DR); label 4: Retinal Detachment (RD); label 5: High Myopia (HM); label 6: Uveitis; label 7: Vitreous Hemorrhage (VH); label 8: Asteroid Hyalosis (AH); label 9: Choroidal Atrophy (CA); label 10: Peripheral Retinal Degeneration (PRD); label 11: Retinal Hole (RH); label 12: Retinitis Pigmentosa (RP); label 13: Normal; label 14: Ocular Albinism Fundus (OAF); label 15: Persistent Hyperplastic Primary Vitreous (PHPV).

We compared the artificial intelligence model with ophthalmologists of different levels. The results showed that the artificial intelligence model demonstrated higher accuracy in all disease diagnosis tasks compared to intermediate and junior ophthalmologists, approaching 1. The accuracy of intermediate ophthalmologists was slightly lower than that of the AI model but still maintained relatively high performance, with accuracy values all above 0.8. Junior ophthalmologists had the lowest accuracy among the three groups, ranging from 0.6 to 0.9. The results are shown in Figure 4.

## Discussion

This study developed a deep learning model integrating DenseNet121 and XGBoost for diagnosing 16 types of retinal diseases in UWF fundus images. The results demonstrated that the model achieved exceptional diagnostic performance on both the validation set and the external test set. On the validation set, for common retinal diseases such as DR, AMD, and RVO, the model exhibited nearly perfect classification accuracy, with AUC values exceeding 0.975 and accuracy rates above 0.980. For rare retinal diseases such as PHPV, OAF, and RP, the model also demonstrated robust diagnostic capabilities, achieving AUC values above 0.970 and accuracy rates above 0.998. On the external test set, the model’s generalization ability was thoroughly validated. For common retinal diseases, AUC values exceeded 0.950 and accuracy rates were above 0.970; for rare retinal diseases, AUC values remained above 0.893 and accuracy rates were above 0.991. Consequently, the method proposed in this study holds significant clinical application potential in the field of intelligent diagnosis of retinal diseases.

It is important to highlight that the model’s superior performance in diagnosing both common and rare diseases does not signify overfitting. First, the complete independence between the training set and the external test set ensures no data leakage occurs (Chicco and Shiradkar, 2023; Gan et al., 2022). Second, the enhanced ability of the model to recognize rare diseases with limited samples can be attributed to the hybrid model architecture, which effectively alleviates the bias arising from class imbalance (Senan et al., 2022). Additionally, Grad-CAM visualization confirms that the regions the model focuses on align well with clinical lesions, thereby reinforcing the medical validity of its diagnostic outcomes (Long et al., 2025; Chen et al., 2021). Consequently, the high accuracy achieved by the model demonstrates its strong generalization capability rather than mere memorization of the training data.

Compared with the single-disease detection models of Ghasemi Falavarjani et al. (2017), Christ et al. (2024), this study, for the first time, realized comprehensive identification of 16 types of retinal diseases within a unified framework. It covered nine common retinal diseases, such as RVO, AMD, DR, RD, HM, Uveitis, VH, PRD, and RH, as well as six rare retinal diseases, including RAO, AH, CA, RP, OAF, and PHPV. The study abandoned the characteristic of traditional end-to-end deep learning models that rely on large-scale labeled data. Instead, it used transfer learning to extract high-level image features with the pre-trained DenseNet121 on ImageNet. Its dense connection structure enhanced the joint capture ability of subtle lesions and wide-field anatomical features through cross-layer feature reuse. Compared with Zhang et al. (2021) who directly fine-tuned large-scale networks such as seResNext50, this strategy reduced the computational load while ensuring the accuracy of feature extraction. The XGBoost classifier allocated weights dynamically to high-dimensional features through the Boosting algorithm, effectively solving the classification bias of the DenseNet pure convolutional network in unbalanced category scenarios (Ashwini and Bharathi, 2024; Seo et al., 2022). This ensured that rare diseases with a sample size of only 4% still achieved high classification performance.

Furthermore, Grad-CAM heat maps reveal that the model’s focus areas are highly consistent with clinical gold standards (e.g., in DR screening, it accurately highlights microaneurysms and hard exudates rather than irrelevant hemorrhages or fibroproliferative foci). Notably, for rare diseases such as PHPV, the activated regions precisely correspond to the vitreous proliferation foci. While Nagasawa et al. (2018) achieved high-precision detection of macular holes using traditional CNN-based processing of ultra-wide-angle fundus images, their model exhibits limitations in complex pathological scenarios. Specifically, an explanation analysis indicates that optical distortions in input data can cause activation regions to shift toward non-lesion areas such as the optic disc. In contrast, the deep learning framework proposed in this study demonstrates significant technical advantages and clinical applicability. This capability provides traceable pathological evidence for AI-assisted decision-making, thereby enhancing clinicians’ trust in diagnostic results.

The model demonstrated outstanding diagnostic performance on both the validation set and the test set, significantly outperforming junior and intermediate doctors. Its core clinical value lies in breaking the excessive reliance on experience in traditional manual reading of films and fundamentally solving the problem of diagnostic inconsistency caused by experience differences. Through advanced technical means, this model effectively compensates for the shortage of ophthalmic medical resources and provides strong support for improving the accessibility and equalization of medical services (Gan et al., 2025a; Zhu et al., 2022).

Certainly, this study has several limitations. While we have demonstrated the efficacy of the hybrid model, its real-time diagnostic performance and economic impact in actual clinical settings require further validation through additional prospective clinical studies. Specifically, its application value in long-term follow-up cohorts for rare diseases, such as PHPV, remains to be fully explored. Although the model exhibits clinically meaningful discrimination capabilities for rare diseases, the variability in its recall rate highlights the need for cautious evaluation of its standalone diagnostic reliability. This discrepancy primarily arises from insufficient feature learning due to the limited availability of rare disease samples (Zuo et al., 2022). Furthermore, the current model’s reliance on a single imaging modality represents a significant constraint. For complex retinal pathologies, the lack of OCT and other multimodal imaging inputs may compromise diagnostic accuracy (Gan et al., 2025b). Future research should focus on multi-center prospective clinical validation, exploration of cross-modal deep learning fusion models, and optimization of data augmentation strategies tailored for rare diseases (Yan et al., 2024).

## Conclusion

In summary, the deep learning model developed in this study enables automatic identification and classification of multiple ophthalmic diseases on UWF devices, demonstrating robust performance. This model holds potential for ophthalmic disease screening and clinical diagnosis, assisting ophthalmologists in making more accurate and efficient diagnoses, thereby enhancing physician diagnostic efficiency and patient care quality.

## Data Availability

The raw data supporting the conclusions of this article will be made available by the authors, without undue reservation.
